# Complexes of Glycolic Acid with Nitrogen Isolated in Argon Matrices. I. Structures and Thermal Effects

**DOI:** 10.3390/molecules24183262

**Published:** 2019-09-07

**Authors:** Iwona Kosendiak, Jussi M.E. Ahokas, Justyna Krupa, Jan Lundell, Maria Wierzejewska

**Affiliations:** 1 Faculty of Chemistry, University of Wroclaw, Joliot-Curie 14, 50-383 Wroclaw, Poland (I.K.) (J.K.); 2 Department of Chemistry, University of Jyvaskyla, P.O.Box 35, 40014 Jyvaskyla, Finland

**Keywords:** hydrogen bond, matrix isolation, carboxylic acid, computational chemistry, vibrational spectroscopy

## Abstract

Molecular complexes between glycolic acid and nitrogen were studied in a low-temperature argon matrix with FTIR spectroscopy, and supported by MP2 and BLYPD3 calculations. The calculations indicate 11 and 10 stable complex structures at the MP2 and BLYPD3 levels of theories, respectively. However, only one hydrogen-bonded complex structure involving the most stable SSC conformer of glycolic acid was found experimentally, where the nitrogen molecule is bound with the carboxylic OH group of the SSC conformer. The complex shows a rich site structure variation upon deposition of the matrix in different temperatures and upon annealing experiments, which provide interesting prospects for site-selective chemistry.

## 1. Introduction

Hydrogen bonding is a significant non-covalent interaction that plays an important role in many areas of biology and chemistry [1,2]. Weakly bound molecular complexes with hydrogen bond or van der Waals interactions are frequently encountered in atmospheric chemistry and are known to affect both chemical and photochemical processes [3,4,5]. In complexes, the electronic, vibrational and rotational levels are disturbed as a result of interaction between complex subunits, and this leads to changes in spectral and photochemical characteristics of the complex components. On the other hand, weakly bound complexes frequently represent shallow energy minima on their potential energy surfaces, which lead to thermodynamic instability of such species at room temperature. From this point of view, the low temperature matrix isolation technique coupled with different spectroscopic methods is a very useful technique to trap and to study weakly bound complexes [6,7]. Recently, it was demonstrated that matrix isolation technique connected with computational studies is a powerful approach to study hydrogen bonds and other intermolecular interactions in complexes involving atmospheric constituents or species related to tropospheric IR-induced chemistry [8,9,10,11,12,13,14].

**Figure 1:**
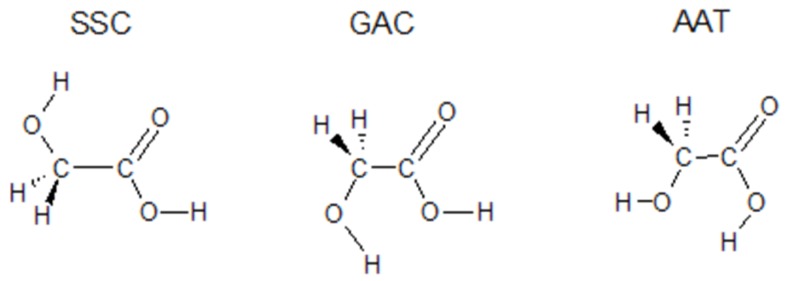
Structures of the three most stable conformers of glycolic acid monomer.

Glycolic acid (GA), the simplest α-hydroxycarboxylic acid with two OH groups, is capable of forming both intra- and intermolecular hydrogen bonds. As such, GA is a model species to study in order to understand how competing OH-groups in the molecule affect the chemical reactivity and capability to form molecular complexes. Several reports on monomeric GA isolated in low-temperature matrices as well as its transformations have appeared in the literature [15,16,17,18,19,20]. The structures of the three most stable GA species are presented in Figure 1. Among the monomer structures, the most stable conformer is the SSC form, which is stabilized by the O–H⋯O intramolecular hydrogen bond. This conformer is present in the gas phase and in low-temperature matrices with an estimated population at room temperature of ca. 95%. A small amount of two less stable GAC and AAT conformers has also been detected [19,20].

Contrary to the monomeric GA, only two studies on GA complexes in low-temperature matrices have appeared in literature. Recently, a study on GA dimers revealed for the first time information on three cyclic GA dimers identified in an argon matrix [21]. All of these dimers were formed between SSC conformers: one dimer with hydrogen bonds between the two carboxylic OH groups, one structure with hydrogen bonds between the two alcoholic OH groups and a mixed structure with one OH group from the carboxylic group acting as proton donor and the alcoholic OH group on the other subunit acting as proton acceptor. Molecular complexes between GA and molecular nitrogen were identified in a study applying Raman spectroscopy for argon-trapped species upon high vibrational excitation experiments [22]. In that study, nitrogen was found to form complexes with two GA conformers (SSC and AAT) in low temperature argon environment. However, this study did not scrutinise the actual molecular complex structures involved but only made conclusions of different GA⋯N_2_ systems involved based on their behaviour upon 532 nm irradiation.

These Raman spectroscopy combined with visible light irradiation experiments acted as a prelude to our study presented here. In this paper, we present results of a combined study employing both theoretical methods and FTIR matrix isolation studies on complexes formed between glycolic acid and nitrogen molecule. The target is to study and to identify the 1:1 complexes GA forms with molecular nitrogen. This research is important in order to understand which type of complexes can be formed, if such species are experimentally detected, and, as these formed complexes are used as precursors for near-infrared irradiation experiments, if their photo-induced chemistry differ from isolated GA monomers. The study on the NIR irradiated GA-N_2_ complexes is reported separately in a following paper taking advantage of the results presented here.

## 2. Experimental and Computational Details

The matrix samples were prepared by passing mixtures of high purity argon (Messer, 5.0) and nitrogen (Messer, 6.0) with the N_2_/Ar ratios of 1/4000 through the glass U-tube with glycolic acid (GA) situated outside the cryostat chamber. Optimizing the deposition temperature and matrix flow rate it was possible to obtain matrices containing nearly exclusively monomeric GA and GA⋯N_2_ complexes of 1:1 stoichiometry. The GA/N_2_/Ar gaseous mixtures were deposited onto a cold CsI window kept at 15 K or 18 K in an APD-Cryogenics (ARS-2HW) closed cycle cryostat. Annealing experiments were performed upon the deposited samples at 33 K. The sample temperature was maintained by a Scientific Instruments 9700 temperature controller equipped with a silicon diode and a resistive heater. FTIR spectra were collected at 10 K in a transmission mode with a 0.5 cm^−1^ resolution using a Bruker IFS 66 Fourier Transform spectrometer equipped with a liquid N_2_ cooled MCT detector.

In order to support the experimental work, computational studies were carried out using the Gaussian16 program package [23]. The calculations were performed for GA:N_2_ 1:1 complexes for the three most stable conformers (SSC, GAC, AAT) at the MP2 [24,25,26,27] and B3LYPD3 [28,29,30,31,32] levels of theory using the 6-311++G(2d,2p) basis set. All geometry optimizations were performed with the Boys-Bernardi full counterpoise method by Dannenberg [33,34]. The topological analysis of the electron density (Atoms-In-Molecules, AIM [35]) was performed at the MP2/6-311++G(2d,2p) level using AIM studio program (Version 12.09.23, Standard) [36]. The harmonic vibrational wavenumbers and infrared intensities were calculated both at MP2 and B3LYPD3 levels for the optimised structures to assist the analysis of the experimental spectra. The computed spectra were used to verify that computed complex structures were stable structures. Interpretation of the infrared spectra is based mainly on the MP2 results and this data is presented in the text.

## 3. Results and Discussion

### 3.1. Structure and Energetics of 1:1 GA⋯N_2_ Complexes

Out of seven theoretically predicted conformers of glycolic acid monomer (GA) the three most stable forms, SCC, GAC and AAT, were detected in low-temperature noble gas matrices [19,20]. The fourth SST conformer was identified only in nitrogen matrices upon near-IR excitation of the most stable SSC conformer [19]. Therefore, exclusive interaction of the SSC, GAC and AAT conformers with nitrogen are considered here since these conformers of monomeric GA are the most plausible to appear in a solid argon environment.

**Figure 2:**
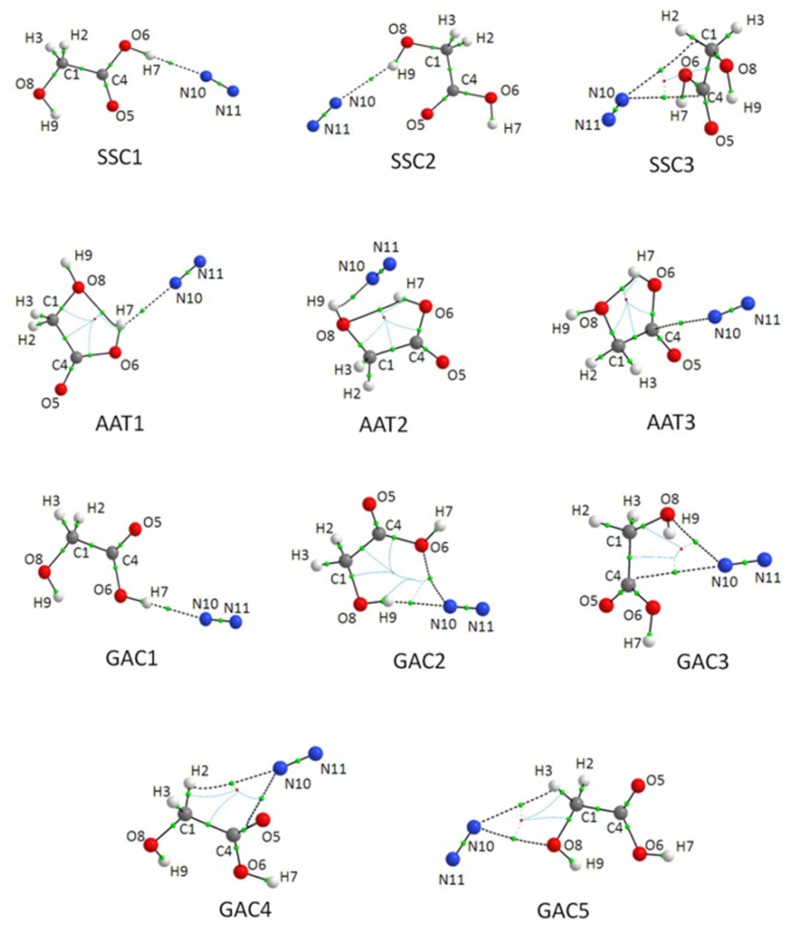
The MP2-optimized 1:1 structures of SSC, AAT and GAC complexes with molecular nitrogen.

At the MP2/6-311++G(2d,2p) level of theory 11 energy minima structures were found for 1:1 GA-N_2_ complexes whereas the calculations performed at the B3LYPD3 level showed the presence of only ten energy minima structures. All optimized GA⋯N_2_ complex structures are shown in Figure 2. The coordinates of all optimised complexes are presented in Table S1 (Supplementary Materials Information).

The AIM approach based on the topological analysis of electron density becomes nowadays almost routine method providing the characteristics of various types of interactions, among them hydrogen bonds and van der Waals interactions [37,38,39]. Two AIM parameters: the electron density ρ(r) and its Laplacian ∇^2^ρ(r) at bond critical points (BCP) obtained for all optimized 1:1 structures at MP2/6-311++G(2d,2p), using the MP2 computed density matrix, are collected in Table 1. Additionally, the positions of the bond (3,−1) critical points derived from the AIM calculations are visualised together with the optimised complex structures in Figure 2.

For interaction of SSC with nitrogen three minima were found and two of them contain the O–H⋯N hydrogen bond formed between carboxylic or alcoholic OH group and N_2_ molecule (species denoted SSC1 and SSC2, respectively). In both of these configurations, the N_2_ molecule lies in the SSC plane. The intermolecular H⋯N distance equals 2.257 and 2.475 Å in SSC1 and SSC2, respectively. Interaction with the carboxylic OH group results in an almost linear hydrogen bond with the Θ(O6H7N10) angle of 179.0° whereas in SSC2 the O–H⋯N bridge is more bent to Θ(O8H9N10) = 152.8°. In the third SSC-N_2_ complex (SSC3) the N_2_ molecule is located above the SSC molecular plane with the N10 atom directed to the centre of the C–C bond. The SSC3 complex also exhibit relatively short N10⋯C1 and N10⋯C4 atomic distances of 3.358 and 3.280 Å, respectively. Based on the AIM analysis two van der Waals interaction paths are found between N10 nitrogen atom and C1 or C4 carbon atoms.

Two hydrogen bonded complexes, analogous to those optimized for SSC⋯N_2_ were found for both GAC⋯N_2_ and AAT⋯N_2_ interactions. The intermolecular H⋯N distances are in the range of 2.262–2.547 Å. Both GAC1 and GAC2 contain almost linear O–H⋯N bridges (178 and 166°, respectively) whereas in AAT1 and AAT2 the hydrogen bond bridges are not linear (in both cases the O–H⋯N angle equals 134°). Similarly to the SSC conformer one non-hydrogen bonded structure (AAT3) was found for AAT. It is characterised by one van der Waals contact between N10 and C1 atoms.

For GAC⋯N_2_ complexes three non-hydrogen bonded minima were found (GAC3, GAC4 and GAC5) but none of them corresponds strictly to the structural configurations found for SSC3 or AAT3. Two of these species are characterised by the C–H⋯N contact and the N10⋯O5 (GAC4) or N10⋯O8 (GAC5) interaction. In the third structure (GAC3) two van der Waals contacts are present between N10⋯O8 and N10⋯C4. We do recognise here that the AIM method does not always result to bond paths with chemical meaning [40,41]. However, here we use the method to give a brief indication how the complex is built up, and how the nitrogen is connected with the GA subunit, even though some of the AIM bond path charts are very complex and can induce multiple interpretation of the nature of the interaction.

The calculated interaction and relative energies of these species optimised at the MP2 and B3LYPD3 levels are summarised in Table 2.

The most stable structure overall in energy is the SSC1 complex. The other two SSC complexes are about 3 kJ mol^−1^ higher in energy. The GAC and AAT complexes with nitrogen molecule are about 10–14 and 12–15 kJ mol^−1^ higher in energy than the complexes of the most stable SSC conformer. These energy differences correspond to the energy differences found for the isolated GA conformers without zero-point nor entropy corrections.

The largest interaction energy among all considered GA⋯N_2_ structures is found for the AAT2 complex, which equals to −9.12 kJ mol^−1^. The interaction in AAT2 is characterised by the alcoholic O–H group acting as a proton donor to nitrogen molecule. As can be seen in Table 2 the interaction energies of all other GA complexes are in the range between −4.02 to −9.12 kJ mol^−1^. Interestingly, both GAC2 and AAT2 complexes with alcoholic OH group interacting with N_2_ are characterised by larger interaction energy than that found for the SSC2 form. The GAC and AAT conformers of the GA monomer were found to be about 9.5 and 12.0 kJ mol^−1^ less stable than SSC whereas, for interaction of N_2_ with the OH alcoholic group, the situation is reversed. Such an observation was described for glyoxylic acid–water complexes [10] as well.

Looking at the AIM results performed for GAC2 the above situation can be related to the presence of a second weak van der Waals interaction between nitrogen molecule and oxygen atom of the carboxylic OH group. In turn, according to the AIM calculations, the intramolecular hydrogen bond, existing already in monomeric AAT, formed between the carboxylic OH and oxygen of the alcoholic OH becomes stronger in AAT2 complex contributing to its overall stability. In general, the formation of an additional van der Waals contact with either of the two OH groups of the GA subunit strengthens the intermolecular interaction. When no such additional interaction appears, a typical hydrogen-bonded structure as found in SSC1 and GAC1 complexes is prevalent in analogy with previous carboxylic acid complexes with nitrogen molecules [42,43].

### 3.2. Computed Spectra

Infrared and Raman spectra were calculated for all the computationally identified complex structures (see Figure 2). The infrared spectra were used to verify that the optimised structures were stable minima on the potential energy surfaces. Additionally, comparison of complex and monomer spectra is useful for understanding the experimental findings. Raman spectra were also computed, and Raman intensities (I_Raman_) were derived from calculated scattering activities values according to the procedure described by Michalska and co-workers [44,45] employing T = 14 K for the Boltzmann distribution factor and excitation frequency of 532 nm (18 797 cm^−1^). These values correspond to the experimental conditions used in our previous work on GA⋯N_2_ [22]. The computational results obtained at the MP2-level of theory are shown in Table 3 for the most stable SSC1 complex. For comparison, the observed Raman bands have been included in Table 3.

The two OH vibrational bands for SSC1 are the alcoholic and carboxylic OH stretching vibrations at 3781 and 3754 cm^−1^, respectively. Another significant vibrational mode is the carbonyl stretching mode (ν_6_) that was computed to be at 1789 cm^−1^. Both of these spectral regions have been probed in the Raman experiments, and GA⋯N_2_ complexes have been identified. In order to make assignment of the complex structures appearing in the experiments, infrared and computational data is needed. Here, we consider all three SSC complexes found computationally in connection with annealing experiments in solid argon. These results are discussed below that shed light on the complex structures also observed in the Raman experiments [22].

### 3.3. Experimental Results

At first, blank experiments were performed for GA isolated in solid argon. The deposition of GA at 15 K yielded matrices containing almost exclusively monomers of the acid. The strong bands of the O–H and the C=O stretching vibrations of the SSC conformer were observed at 3561 and 1773 cm^−1^. Much weaker absorptions of the two less abundant AAT and GAC conformers were barely observed at 3671, 3473 and 1806 cm^−1^ and 3648, 3561 and 1784 cm^−1^ for AAT and GAC, respectively, in accordance with the predicted gas phase abundances. When the deposition temperature was higher than 15 K or when the matrix with GA monomers was annealed a number of new weak bands appeared due to the GA dimers [21].

Complex formation between glycolic acid and nitrogen was observed when GA was deposited together with the N_2_ doped argon with the N_2_:Ar ratio of 1:4000 or higher. New bands due to the N_2_ complexes with GA appeared in several spectral regions in the vicinity of the GA monomer absorptions. Table 4 contains the most characteristic wavenumber shifts (Δν = ν_complex_ − ν_monomer_) calculated for the 1:1 species of interest compared to the experimental values. From now on the notation OH_C_ and OH_A_ denotes carboxylic and alcoholic OH group, respectively.

**Figure 3:**
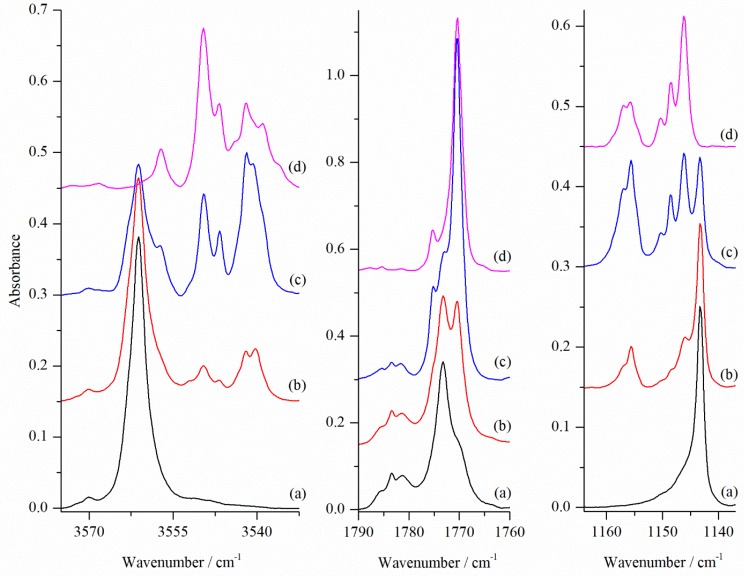
The νOH, δOH_C_ and νC=O regions of the infrared spectra of GA co-deposited with N_2_/Ar = 1/4000 at 15 K, 18 K and 25 K (measurement at 10 K) (traces (**b**–**d**), respectively, compared with GA/Ar spectrum (**a**).

Figure 3 shows the stretching νOH, νC=O and in-plane-deformation δOH_C_ regions of the infrared spectra of GA/N_2_/Ar matrices deposited at three different temperatures and compared to the GA/Ar spectrum. In the νOH stretching region of the spectrum of GA co-deposited at 15 K with the N_2_/Ar = 1/4000 mixture several new maxima due to the GA⋯N_2_ complexes are distinguished. They are situated below the νOH of the SSC conformer at 3549.5, 3546.5 and 3542.0/3540.0 (a doublet) cm^−1^. Additionally, a broadening on the lower wavenumber side of the νOH of the SSC monomer was found at 3556 cm^−1^ (at 15 K seen as a weak shoulder and better shaped at 18 and 25 K). The same set of bands (being much weaker) was observed in the νOH region when GA was deposited with an extremely diluted N_2_/Ar (in the case when small leak in the vacuum system was present). This indicates that all mentioned spectral features are due to the 1:1 GA⋯N_2_ species.

As shown in Figure 3, when matrices were deposited at 15 K and 18 K the most intense of all new bands present in the νOH_C_ region was a doublet at 3542.0/3540.0 cm^−1^. Each of the bands observed in the νOH_C_ region has its counterpart in the in-plane-bending δOH_C_ region. Here, in the region of 1140–1160 cm^−1^, where we find the SSC monomer band at 1143.5 cm^−1^, a new set of bands due to the GA⋯N_2_ complex appeared at higher wavenumbers. A doublet in the νOH_C_ region at 3542.0/3540.0 cm^−1^ and a weak band at 3557.0 cm^−1^ (shifted by −19/−21 cm^−1^ and −4 cm^−1^) fit well to the calculated positions of the stretching vibrations of carboxylic and alcoholic groups, respectively, in the most stable SSC1 complex. The calculated ΔνOH shifts in SSC1 form equal to −31 and −5 cm^−1^ for the hydrogen bonded carboxylic hydroxyl group and for non-hydrogen bonded alcoholic OH, respectively. Similar agreement was also found in other spectral regions as presented in Table 4. For the in-plane-deformation δOH_C_ mode the most intense doublet in the spectra of matrices deposited at 15 and 18K at 1157.0/1155.5 cm^−1^ is blue-shifted by 13.5 and 12 cm^−1^ relative to the corresponding monomer absorption. This is also in a very good agreement with the predicted shift of 15 cm^−1^.

Both in νOH_C_ and δOH_C_ regions additional bands are present, and they are slightly shifted compared with those already assigned above. There are several possible reasons for the observed additional absorptions in the spectra. One of them is that there exists different structures of SSC⋯N_2_ complexes. As shown in Table 4 the νOH shifts predicted for SSC2 and SSC3 complexes do not fit to any of the observed bands. This indicates that there is only one type of complex in the experimental conditions used instead of three different SSC complexes identified computationally. Consequently, only the SSC1 structure is to be formed in the argon matrix, and another explanation should be found for the additional bands in the νOH_C_ and δOH_C_ regions. The most obvious explanation is the presence of multiple trapping sites in the matrix, which can slightly perturb the structures of the complexes. For the SSC1 structure, the position of the N_2_ molecule relative to the OH group of the carboxylic moiety is most susceptible to changes induced by the environment. Depending on the local structure of the surrounding argon atoms, the hydrogen-bonded N_2_ tail is either bent or elongated compared to the most favourable site structure.

**Figure 4:**
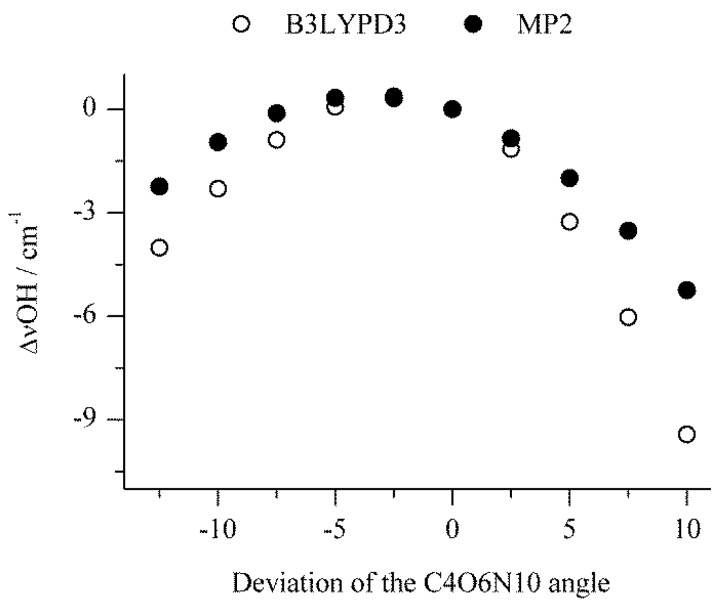
Plot showing changes of the νOH value calculated for each point of the relaxed potential energy scan for the bending movement of the N_2_ molecule in SSC1 complex calculated at the B3LYPD3 and MP2 levels with 6-311++G(2d,2p) basis set versus deviation of the C4O6N10 angle. The zero value of this angle corresponds to the global minimum value.

This idea of perturbed complex structures giving different spectral features was tested with a relaxed potential energy scan for the bending motion of the N_2_ molecule in the hydrogen-bonded SSC1 complex. For each step the C4O6N10 angle describing a position of N_2_ relative to the GA was fixed at values slightly different than that obtained for the global SSC1 minimum (the C4O6N10 angle equals to 104.1 degrees) and all remaining parameters were optimized. The range of angle changes was between 94.0 and 119.0 degrees with a step of 2.5 degrees. Figure 4 shows the changes of the νOH wavenumber calculated for each point of the relaxed potential energy scan. The relative electronic energy of the SSC1 complex and the corresponding changes of the νC=O and δOH_C_ wavenumbers are presented in Figure S1 (Supplementary Materials Information). All points presented in Figure 4 and Figure S1 represent structures that all have positive wavenumbers in their computed spectra. Accordingly, the bending movement of the N_2_ molecule in the studied range results in very small changes in energy indicating that the potential energy surface is relatively flat. The changes of the wavenumbers of the vibrations depend on how much the nitrogen molecule is averted from its equilibrium position. The highest deviations of the νOH_C_ and δOH_C_ wavenumbers are between 5 and 9 cm^−1^ whereas that obtained for νC=O is smaller and equals ca. 2 cm^−1^. The predicted values of the ΔνOH_C_ and ΔδOH_C_ are of the same order as differences in positions of the components of the absorption in the experimental OH stretching and in-plane deformation regions. Therefore, it is plausible to assume that the additional features observed in the spectra are due to the SSC1 complex in different local structures (sites) in the matrix.

In the middle panel of Figure 3, an intense new feature appeared upon complex formation in the νC=O region. The intense band at 1770.5 cm^−1^ is red shifted by 2.5 cm^−1^ compared to the νC=O of SSC monomer (1773 cm^−1^). Its position and intensity are consistent with those predicted computationally for the νC=O in SSC1 structure (see Table 4). No site splitting in the ΔνC=O region was detected in agreement with the small wavenumber changes predicted for this mode upon N_2_ movement (see Figure S1). This is also in accordance with the explanation of site structures observed for the OH stretching vibrations, which are due to the movement of the nitrogen molecule in the hydrogen-bonded bridge. The νC=O mode is insensitive to these changes.

**Figure 5:**
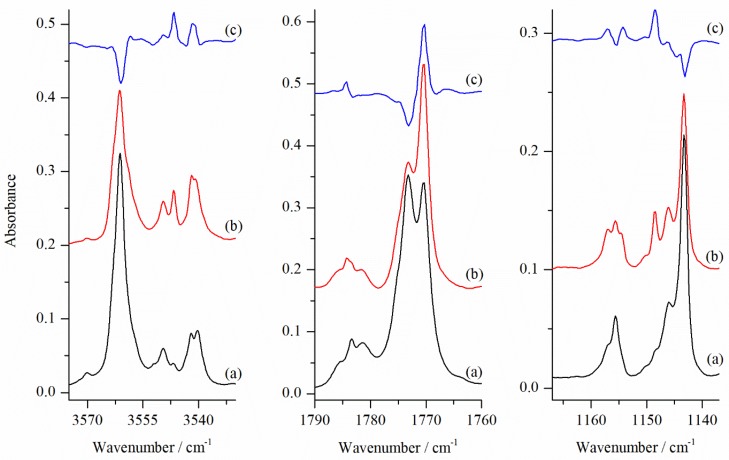
The νOH, δOH_C_ and νC=O regions of the infrared spectra of GA co-deposited with N_2_/Ar = 1/4000 (**a**) at 15 K and (**b**) after annealing at 33 K (measurement at 10 K) and (**c**) the corresponding difference spectrum (**b**) minus (**a**). The bands at 3561.0, 1773.0 and 1143.0 cm^−1^ belong to the SSC monomer.

Figure 5 shows the influence of the matrix annealing in the νOH, νC=O and δOH_C_ regions of the GA/N_2_/Ar matrices deposited at 15 K. The difference spectra shown in the upper part of the figure indicate that the components of the ν OH absorption were differently affected by rising temperature to 33 K. Combining the information from Figure 4 and Figure 5 indicates that deposition of the matrix at different temperatures favour different local structures of the SSC1 complex. Annealing of the matrix influences the site structure very little. In the OH stretching region, there appear mainly two sites (corresponding to doublet at 3542 and 3540 cm ^−1^, and a band at 3546.5 cm^−1^) which yield hydrogen-bonded complexes from close contact pairs between nitrogen and GA. The third site, evidenced by a band at 3549.5 cm^−1^, appears in deposition but it is not growing upon annealing.

## 4. Conclusions

Matrix isolation FTIR spectroscopy and computational chemistry have been employed to study glycolic acid complexes with a nitrogen molecule in an argon matrix. Computationally, at the MP2 level, 11 different GA⋯N_2_ complex structures were obtained for the SSC, GAC and AAT glycolic acid monomers. For SSC and GAC the largest interactions between the complex subunits were found for structures, where the nitrogen molecule forms a linear hydrogen-bonded structure with the GA subunit. In the case of AAT a cyclic structure involving both OH groups appeared to be the most tightly bound instead of the linear hydrogen-bonded structure.

In the experimental conditions used, the glycolic acid molecule is prevalently complexed with one nitrogen molecule. Deposition of the matrix in different temperatures indicate that there are three different local site structures of the complex formed. Annealing experiments are helpful in distinguishing the different sites from each other, and here two of the three sites were found to be further enhanced from local near contact pairs. The existence of site structures of one complex is also implied by the computational methods based on the vibrational shifts computed for the complexes. Additionally, these sites are stable enough that they could not be interconverted upon annealing experiments.

In conjunction with recent Raman spectroscopy study [22] and the results obtained here by FTIR and computational methods, it can be concluded that co-depositing GA and N_2_ in a large excess argon matrix produces only hydrogen bonded SSC⋯N_2_ complexes occupying three different local matrix sites. In order to gain insight on the local structures and their interconversion dynamics upon annealing molecular dynamics simulations of the doped matrices and irradiation experiments should be performed. The latter approach is employed in the following paper [46], where the site-selective chemistry of SSC⋯N_2_ is be used to unravel some structural features of the sites and their impact on the photo-induced processes.

## Figures and Tables

**Table 1:** Interatomic distances (Å), angles (degree) and electron density parameters of the bond (au) of the SSC, GAC and AAT complexes with N_2_ (1:1) computed at the MP2/6-311++G(2d,2p) level.

Complex	Intermolecular Parameters			AIM Parameters		
	Interatomic Distances		Angle	BCP	ρ (r)	∇^2^ρ(r)
	H⋯Y	X⋯Y	X–H⋯Y			
SSC1	2.257	3.226	179.0	H7⋯N10	0.014	0.053
SSC2	2.475	3.363	152.8	H9⋯N10	0.009	0.037
SSC3		3.358		C1⋯N10	0.006	0.024
		3.280		C4⋯N10	0.006	0.024
	Ring critical point				0.006	0.025
GAC1	2.262	3.230	178.1	H7⋯N10	0.014	0.053
GAC2	2.363	3.304	166.4	H9⋯N10	0.012	0.045
		3.410		O6⋯N10	0.004	0.016
	Ring critical point				0.004	0.016
GAC3		3.249		O8⋯N10	0.006	0.022
		3.252		C4⋯N10	0.006	0.026
	Ring critical point				0.005	0.025
GAC4		3.323		O5⋯N10	0.006	0.023
	2.971	3.684	123.1	H2⋯N10	0.005	0.017
	Ring critical point				0.005	0.018
GAC5	2.832	3.521	121.5	H3⋯N10	0.004	0.016
		3.188		O8⋯N10	0.005	0.018
	Ring critical point				0.004	0.018
AAT1	2.459	3.212	134.2	H7⋯N10	0.010	0.040
	1.950	2.584	120.7	H7⋯O8	0.027	0.117
	Ring critical point				0.025	0.143
AAT2	2.547	3.287	133.9	H9⋯N10	0.008	0.030
	1.985	2.640	122.7	H7⋯O8	0.028	0.108
	Ring critical point				0.026	0.137
AAT3	1.922	2.575	122.2	H7⋯O8	0.029	0.122
		3.184		C4⋯N10	0.006	0.027
	Ring critical point				0.026	0.151

**Table 2:** Computed interaction energies and relative energies compared to global minimum SSC1 (in kJ mol^−1^).

Structure	Interaction Energy		Relative Energy	
	MP2	B3LYPD3	MP2	B3LYPD3
SSC1	−7.70	−8.41	0.00	0.00
SSC2	−4.48	−5.15	3.23	3.27
SSC3	−5.40	−5.77	2.35	2.63
GAC1	−7.53	−8.28	10.70	10.49
GAC2	−5.61	−6.23	12.80	12.68
GAC3	−5.02	−5.31	13.28	13.46
GAC4	−4.48	−4.94	13.75	13.78
GAC5	−4.02	-	14.21	-
AAT1	−4.85	−5.56	15.71	16.23
AAT2	−9.12	−9.71	12.19	12.34
AAT3	−5.40	−5.73	15.08	15.91

**Table 3:** Computed band positions for the most stable SSC^⋯^N_2_ complex (SSC1) compared to the experimental Raman band positions (cm^−1^) together with computed infrared (km mol^−1^) and Raman intensities.

Mode	Band Position	I_IR_	I_Raman_	Raman Exp [22]
ν_1_	3781	79	477	
ν_2_	3754	302	1880	3562, 3554, 3545
ν_3_	3130	7	1202	
ν_4_	3087	25	2536	
ν_5_	2175	1	674	
ν_6_	1789	246	739	1777, 1775
ν_7_	1518	12	509	
ν_8_	1492	2	106	
ν_9_	1372	124	202	
ν_10_	1315	33	134	
ν_11_	1271	0	355	
ν_12_	1190	161	175	
ν_13_	1112	226	168	
ν_14_	1053	1	13	
ν_15_	878	28	1107	
ν_16_	689	108	16	
ν_17_	653	18	518	
ν_18_	540	1	204	
ν_19_	480	18	528	
ν_20_	340	77	7	
ν_21_	287	12	32	
ν_22_	102	0	1504	
ν_23_	92	8	1787	
ν_24_	80	5	757	
ν_25_	79	2	224	
ν_26_	23	0	387	
ν_27_	18	1	2940	

**Table 4:** MP2/6-311++G(2d,2p) calculated wavenumber shifts Δν (cm^−1^) and intensities (km mol^−1^) of the SSC complexes ^a^ with nitrogen compared to the corresponding experimental shifts.

MP2/6-311++G(2d,2p)						Experimental Shifts	Assignment
SSC1		SSC2		SSC3 *			
Δν	I	Δν	I	Δν	I	Δν	
−5	79	3	119	0	32	−4	(ν_1_) νOH_A_
−31	302	1	117	0	140	−11.5, −14.5, −19.0/−21.0	(ν_2_) νOH_C_
−5	246	1	274	−1	249	2, −3	(ν_6_) νC=O
10	124	−4	126	1	123	12, 6	(ν_9_) δOH_A_ + δOH_C_ + νC–O_C_
15	161	−2	126	0	139	14, 13, 8, 5, 3	(ν_12_) δOH_C_ + νC–O_C_ +ωCH_2_
4	28	−1	30	1	28	6, 4, 2	(ν_15_) νC–C + νC–O_C_

^a^ Corresponding positions for the SSC monomer are: 3786, 3785, 1794, 1362, 1175 and 874 cm^−1^ (MP2/6-311++G(2d,2p)) and 3561, 3561, 1773, 1332, 1143 and 854 cm^−1^ (Ar matrix [19]). * In SSC3 the νOH_C_ and νOH_A_ vibrations are coupled.

## Supplementary Materials

The following are available online, Figure S1. Plot showing changes of the energy, νC=O, δOH wavenumbers calculated for each point of the relaxed potential energy scan for the bending movement of the N_2_ molecule in SSC1 complex calculated at the B3LYPD3 and MP2 levels with 6-311++G(2d,2p) basis set versus deviation of the C4O6N10 angle. Table S1**.** Cartesian coordinates of N_2_, GA monomers and GA⋯N_2_ complexes of the 1:1 stoichiometry calculated at MP2/6-311++G(2d,2p) level.
