# Supplementary material for: Complexes of Glycolic Acid with Nitrogen Isolated in Argon Matrices. I. Structures and Thermal Effects
Source: Molecules. 2019 Sep 7;24(18):3262. doi: 10.3390/molecules24183262 (PMC6767210; doi:10.3390/molecules24183262)
Supplement: Supplementary file 1 [file molecules-24-03262-s001.pdf]

# Supporting Information

## Complexes of glycolic acid with nitrogen isolated in argon matrices. I. Structures and thermal effects.

Iwona Kosendiak, Jussi Ahokas, Justyna Krupa, Jan Lundell, Maria Wierzejewska

Table S1. Cartesian coordinates of $N_2$, GA monomers and GA···$N_2$ complexes of the 1:1 stoichiometry calculated at MP2/6-311++G(2d,2p) level.

SSC
C 0.73300100 -0.75124500 0.00000800
H 0.70795700 -1.39240000 -0.88055400
H 0.70795700 -1.39237900 0.88058600
C -0.51158500 0.09934900 -0.00000100
O -0.49686000 1.31106000 -0.00000600
O -1.63678200 -0.64330000 0.00000100
H -2.37993300 -0.02471000 -0.00000200
O 1.89019500 0.05154400 -0.00000400
H 1.58309600 0.96644300 0.00000000

SSC1
C -2.19728600 0.51298200 0.00003100
H -2.39454400 1.12362800 -0.88048300
H -2.39449800 1.12359300 0.88058100
C -0.73575500 0.14192300 -0.00001600
O -0.33819900 -1.00416900 -0.00003800
O 0.06556200 1.22243400 -0.00002400
H 0.97950100 0.90161700 -0.00005200
O -3.00771600 -0.63929300 0.00002000
H -2.40215700 -1.39098100 -0.00002100
N 3.09629900 0.11824400 -0.00000400
N 4.05409600 -0.44953800 0.00003600

SSC2
C -1.40901000 1.10190500 0.00000300
H -1.97522000 1.40619800 0.88001900
H -1.97520400 1.40621000 -0.88001800
C -1.31572500 -0.40251800 -0.00000600
O -0.26996400 -1.01441900 0.00000400
O -2.53226100 -0.98580800 -0.00002300
H -2.38319200 -1.94109100 -0.00002500
O -0.13199800 1.69324100 0.00001800
H 0.51174200 0.97416600 0.00002000
N 2.82382400 0.08978000 0.00001100
N 3.69675800 -0.60205300 -0.00000700

SSC3
C 0.88502500 0.91815700 -0.65295900
H 0.29241500 0.68541800 -1.53532700
H 1.86705700 1.25653200 -0.98531700
C 1.07351000 -0.34762900 0.14278800
O 0.77841900 -0.45444200 1.31361600
O 1.61877000 -1.33666000 -0.59249300
H 1.71532400 -2.09969400 -0.00654500
O 0.24270000 1.90498500 0.12101100
H 0.19090400 1.55107400 1.01733700
N -2.15490900 -0.48995900 -0.41950100
N -3.12166600 -0.32826500 0.11004500

GAC
C 0.60626000 -0.10750700 0.00588700
O 1.62725600 -0.73987700 -0.12167100
O 0.56755600 1.24936000 0.06943900
H 1.48170000 1.55321100 -0.01314000
C -0.76829200 -0.71545300 0.18325400
H -0.81118400 -1.61268000 -0.42246500
H -0.84426600 -1.01334100 1.23187900
O -1.84011500 0.11800500 -0.20953300
H -1.69164000 0.99067200 0.16300000

GAC1
C 2.24091200 0.40811100 0.12766500
H 2.49570700 0.67915900 1.15522000
H 2.62307400 1.18291100 -0.52624300
C 0.73046400 0.43248400 0.03690500
O 0.06740000 1.43670700 -0.07767000
O 0.19960700 -0.80922700 0.16162400
H -0.76318400 -0.70857800 0.13102900
O 2.83760400 -0.81411000 -0.25888100
H 2.33083200 -1.52836900 0.13557800
N -3.00237600 -0.41198000 0.01449800
N -4.04784900 -0.04168400 -0.08358200

GAC2
C -1.25133700 1.09484000 0.27362500
H -1.19675500 1.22296200 1.35769500
H -2.10305700 1.66104700 -0.08402900
C -1.54556500 -0.36998300 0.02691100
O -2.63953700 -0.87418000 0.12443700
O -0.42066900 -1.07506700 -0.25973000
H -0.70042600 -1.99490300 -0.36373600
O -0.10425900 1.59261500 -0.38061600
H 0.64554300 1.03789500 -0.14965600
N 2.77544400 0.05222400 0.12158300
N 3.71767500 -0.54123700 0.10181500

GAC3
C -0.88958500 0.94029200 -0.66457500
H -1.87426700 1.35105100 -0.89965800
H -0.35558800 0.80200500 -1.59733500
C -1.12741000 -0.43126400 -0.07048400
O -1.46840600 -1.40059400 -0.70463800
O -0.97550700 -0.43409500 1.28004100
H -1.19037500 -1.32969200 1.57458400
O -0.12742900 1.81875300 0.13831900
H -0.47230500 1.78049800 1.03373300
N 3.10964900 -0.30392800 0.13194400
N 2.11395700 -0.48615000 -0.33347900

GAC4
C -1.21558500 -0.69772900 -0.60783300
H -0.40364000 -1.42740900 -0.56857500
H -1.54285400 -0.61394900 -1.63731200
C -0.61379400 0.63138800 -0.20801100
O 0.04220500 1.33661400 -0.93761200
O -0.84405400 0.91660800 1.09984500
H -0.39824600 1.75563800 1.27889100
O -2.33024900 -1.10050200 0.16275000
H -2.10607700 -0.99267900 1.09033900
N 3.41105100 -0.35151500 0.07845000
N 2.37236000 -0.72638700 0.22610000

GAC5
C -0.42366900 -0.10657300 0.88979300
H -0.85642600 -0.31180600 1.87189500
H 0.23411700 0.74893300 0.98595100
C -1.57056000 0.29394700 -0.01260100
O -2.08864400 1.38484000 -0.02312400
O -1.99730600 -0.74591500 -0.77665600
H -2.74935900 -0.41807900 -1.28832000
O 0.35177100 -1.19012600 0.41620200
H -0.24787300 -1.88627400 0.13620800
N 3.61529700 0.03429300 -0.52995900
N 2.87875300 0.70179100 -0.02722200

AAT
C 0.66016200 -0.04572800 0.00278000
O 1.71427600 -0.62945000 -0.02103700
O 0.56522300 1.29611000 0.02446000
H -0.38101700 1.51035300 0.03381100
C -0.66740800 -0.78879800 0.02744000
H -0.66700600 -1.49915900 -0.79497000
H -0.71456200 -1.33538200 0.96647700
O -1.73920000 0.14908500 -0.09519200
H -2.51633800 -0.19461500 0.34751300

AAT1
C -1.52501000 -0.47699900 -0.00155700
O -2.53936300 -1.12595000 -0.06602300
O -0.30522700 -1.03379300 0.08751700
H 0.34601400 -0.31494100 0.12015200
C -1.55104100 1.04395300 0.00061700
H -2.13859500 1.36686500 -0.85490600
H -2.05505100 1.35625600 0.91255300
O -0.21434900 1.54405500 -0.06474500
H -0.17885500 2.40888700 0.34621800
N 2.80262800 -0.22176300 0.06563600
N 3.90513000 -0.24870800 -0.09026100

AAT2
C 1.01259700 -0.61659000 -0.04040500
O 1.07474200 -1.66787900 -0.62713900
O 0.96369800 -0.52957800 1.30443800
H 0.91759600 0.41848100 1.51030700
C 1.01061700 0.72895900 -0.75652200
H 2.01586700 0.91121700 -1.12453400
H 0.34191900 0.66179900 -1.60918200
O 0.67725600 1.80957400 0.11500800
H -0.27826200 1.90764000 0.10539600
N -2.22188000 0.26976000 -0.05738600
N -3.04411500 -0.47980200 -0.00531100

AAT3
C 0.70318500 -0.76748400 -0.02516400
O 0.34235600 -1.75918200 -0.60776400
O 0.64797500 -0.64440900 1.31368600
H 0.98833300 0.23912900 1.52665500
C 1.26811200 0.43681500 -0.76200200
H 2.19500900 0.12250100 -1.23619800
H 0.55296300 0.71963600 -1.52933900
O 1.48222700 1.50386800 0.16557000
H 2.21466200 2.04412500 -0.13388900
N -2.17333400 0.58100400 -0.23200400
N -3.19226800 0.28419800 0.10683700

$N_2$
N 0.00000000 0.00000000 0.55697400
N 0.00000000 0.00000000 -0.55697400

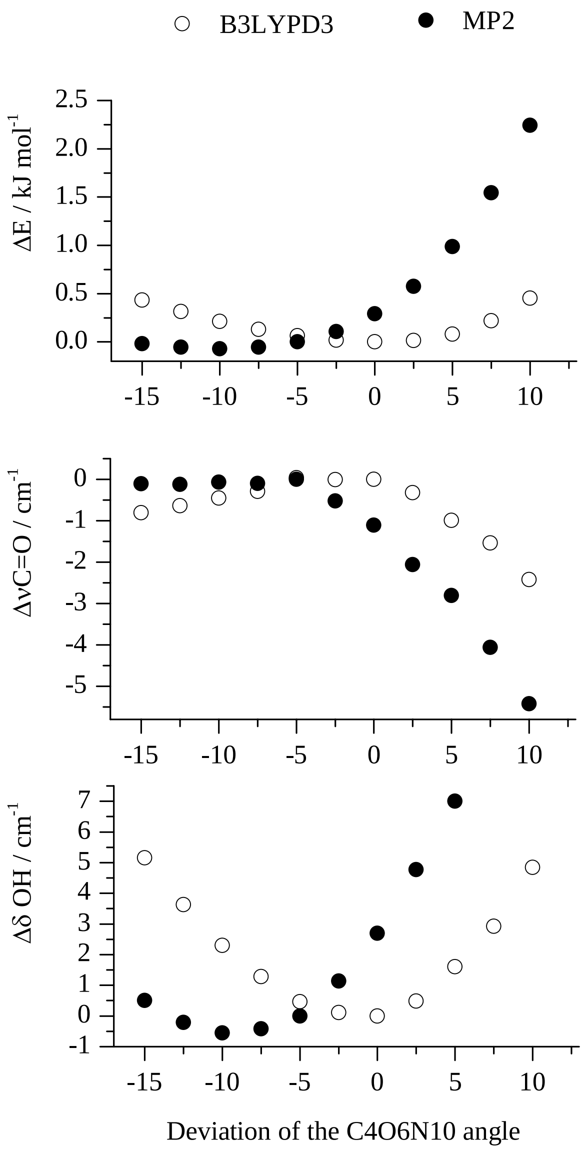


Fig. S1. Plot showing changes of the energy, νC=O, δOH wavenumbers calculated for each point of the relaxed potential energy scan for the bending movement of the $N_2$ molecule in SSC1 complex calculated at the B3LYPD3 and MP2 levels with 6-311++G(2d,2p) basis set versus deviation of the C4O6N10 angle .ΔE (in kJ $mol^{-1}$) is the energy difference between the values obtained for the global minimum and each partially optimized geometry;The zero value of this angle corresponds to the global minimum value.
